# Effect of Antimicrobial Denture Base Resin on Multi-Species Biofilm Formation

**DOI:** 10.3390/ijms17071033

**Published:** 2016-06-29

**Authors:** Keke Zhang, Biao Ren, Xuedong Zhou, Hockin H. K. Xu, Yu Chen, Qi Han, Bolei Li, Michael D. Weir, Mingyun Li, Mingye Feng, Lei Cheng

**Affiliations:** 1 State Key Laboratory of Oral Diseases, Sichuan University, Chengdu 610041, China; zalmancoco@163.com (K.Z.); renbiao@scu.edu.cn (B.R.); zhouxd@scu.edu.cn (X.Z.); hanqi992011@163.com (Q.H.); libolei@stu.scu.edu.cn (B.L.); limingyun@scu.edu.cn (M.L.); 2 Department of Operative Dentistry and Endodontics, West China Hospital of Stomatology, Sichuan University, Chengdu 610041, China; 3 Biomaterials & Tissue Engineering Division, Department of Endodontics, Prosthodontics and Operative Dentistry, University of Maryland Dental School, Baltimore, MD 21201, USA; Hxu@umaryland.edu (H.H.K.X.); MWeir@umaryland.edu (M.D.W.); 4 Department of Oral Pathology, West China Hospital of Stomatology, Sichuan University, Chengdu 610041, China; chen_yu55@hotmail.com

**Keywords:** denture base resin, dimethylaminododecyl methacrylate, antimocrobial material, inter-kingdom biofilm, biocompatibility

## Abstract

Our aims of the research were to study the antimicrobial effect of dimethylaminododecyl methacrylate (DMADDM) modified denture base resin on multi-species biofilms and the biocompatibility of this modified dental material. *Candida albicans* (*C. albicans*), *Streptococcus mutans* (*S. mutans*), *Streptococcus sanguinis* (*S. sanguinis*), as well as *Actinomyces naeslundii* (*A. naeslundii*) were used for biofilm formation on denture base resin. Colony forming unit (CFU) counts, microbial viability staining, and 2,3-bis(2-methoxy-4-nitro-5-sulfophenyl)-2H-tetrazolium-5-carboxanilide (XTT) array were used to evaluate the antimicrobial effect of DMADDM. *C. albicans* staining and Real-time PCR were used to analyze the morphology and expression of virulence genes of *C. albicans* in biofilm. Lactate dehydrogenase (LDH) array and Real-time PCR were conducted to examine the results after biofilm co-cultured with epithelial cell. Hematoxylin and eosin (HE) staining followed by histological evaluation were used to study the biocompatibility of this modified material. We found that DMADDM containing groups reduced both biomass and metabolic activity of the biofilm significantly. DMADDM can also inhibit the virulence of *C. albicans* by means of inhibiting the hyphal development and downregulation of two virulence related genes. DMADDM significantly reduced the cell damage caused by multi-species biofilm according to the LDH activity and reduced the expression of *IL-18* gene of the cells simultaneously. The in vivo histological evaluation proved that the addition of DMADDM less than 6.6% in denture material did not increase the inflammatory response (*p* > 0.05). Therefore, we proposed that the novel denture base resin containing DMADDM may be considered as a new promising therapeutic system against problems caused by microbes on denture base such as denture stomatitis.

## 1. Introduction

Denture stomatitis, a common multi-factorial disease, can be caused by biofilm of denture surface owing to insufficient hygiene of oral cavity and dentures [1]. *Candida* associated denture stomatitis, also called chronic actrophic candidiasis, affects 11% to 67% of geriatric complete denture wearers [2,3,4]. Denture wearers also have a higher risk of dental decay and periodontal diseases in the residual teeth [5,6]. Moreover, denture biofilm was also associated with systemic diseases, such as malodor, aspiration pneumonia, pulmonary candidiasis, as well as infectious endocarditis, especially in aged denture wearers [7,8,9,10].

These concerns suggest the need for a novel modified denture material, especially the ones with antimicrobial activity. Lots of strategies had been applied to reduce the potential bacterial or fungal pathogens, such as denture cleaning technologies based on denture cleaners [11], and denture material antimicrobial modification [12]. Recently, a lot of drugs were added to the denture base to carry out antimicrobial modification with basic antimicrobial categories of non-release antimicrobial agents, polymeric surface coatings, and germifuga-releasing polymers, and with antimicrobial mechanisms of electrostatic repulsion, contact or releasing active biocide [13,14,15,16,17]. *C. albicans* is known as a kind of opportunistic pathogen which can be frequently isolated from oral mucosal surfaces. Its virulence-associated factors include phospholipases, secreted aspartyl proteinases, adhesions, as well as morphogenesis. However, previous modified denture base only evaluated the anti-*C. albicans* single species biofilm, due to the strong association of *C. albicans* with denture stomatitis development, while neglected the bacterial influence [18,19]. In fact, bacteria can even promote the pathogenicity of *Candida* and cause the co-infections [20,21,22]. It is more valid to construct multi-species biofilm containing denture stomatitis associated bacteria and fungi to value the effects of antimicrobial modified denture materials in vitro.

Dimethylaminododecyl methacrylate (DMADDM), a new kind of quaternary ammonium salts (QAS), is known as a long-lasting and remarkable antibacterial additive with well biocompatibility, which had been incorporated into many dental materials [23,24,25,26,27]. QAS had a “contact killing” antibacterial mechanism, as the QAS immobilized materials surfaces were highly positively charged, which can attract the negatively charged bacteria, furthermore, the bacteria membrane would be penetrated and interrupted by the long fatty alkyl chains of QAS [28,29]. In this study, we created a kind of denture based on heat-polymerized denture-base resins in addition to DMADDM and evaluated their effect on inter-kingdom biofilm constituted of fungi and bacteria in vitro, Finally, we assessed the biocompatibility of this new modified material in rats.

## 2. Results

### 2.1. Dimethylaminododecyl Methacrylate (DMADDM) Reduced the Viable Microbes in the Multi-Species Biofilms

**Figure 1:**
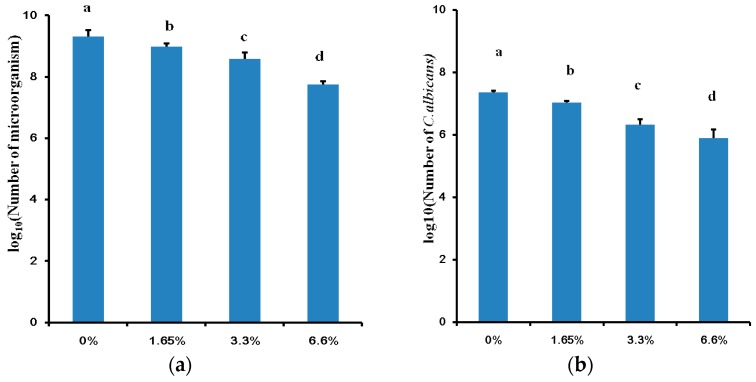
Colony forming unit (CFU) counts of multi-species biofilms: (**a**) The total CFU counts of 72 h multi-species biofilms in different dimethylaminododecyl methacrylate (DMADDM) containing groups; (**b**) The *C. albicans* CFU counts of 72 h multi-species biofilms in different group. Values are significantly different when labelled with different letters (*p* < 0.05).

After 72 h, 1.65% DMADDM, 3.3% DMADDM, and 6.6% DMADDM significantly reduced the total viable microbes of the multi-species biofilms (Figure 1a) and the amount of *C. albicans* (Figure 1b) according to the CFU counting assay (*p* < 0.05). The total microbes and *C. albicans* CFU were reduced more than 53% in 1.65% DMADDM group, and reduced more than 80% to 90% in 3.3% DMADDM group, while reduced about 97% in 6.6% DMADDM groups when compared with control group suggesting the antibacterial and antifungal abilities of DMADDM.

### 2.2. DMADDM Changed the Multi-Biofilm Structuel and Inhibited Themetabolic Abilities

**Figure 2:**
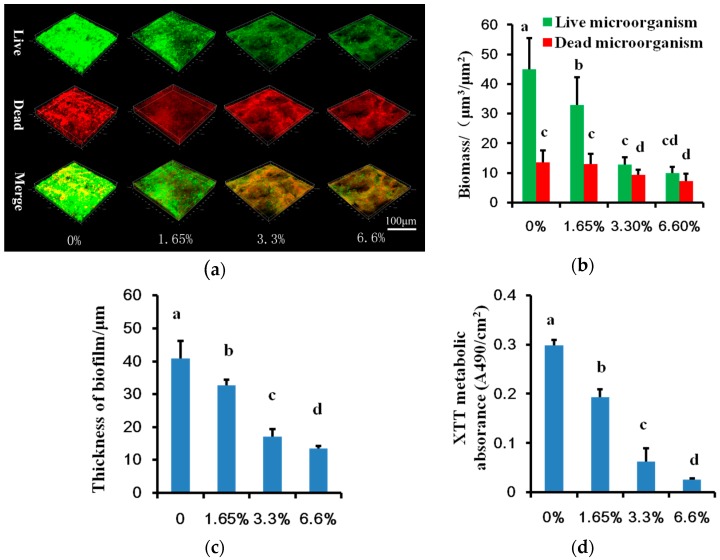
Biofilm structural and metabolic analyses: (**a**) The 3D reconstruction of 72 h multi-species biofilm in different DMADDM containing groups, live microbes dyed green, dead microbes dyed red, adjacent live and dead microbes were presented as yellow when they were merged; (**b**) The biomass of 72 h multi-species biofilm; (**c**) The thickness of 72 h multi-species biofilms in different DMADDM containing groups; (**d**) The 2,3-bis(2-methoxy-4-nitro-5-sulfophenyl)-2H-tetrazolium-5-carboxanilide (XTT) results of multi-species biofilms formed on different DMADDM containing denture base resin. Values are significantly different when labelled with different letters (*p* < 0.05).

In three-dimensional biofilm CLSM (confocal laser scanning microscope) images, live microbes dyed green, dead microbes dyed red (Figure 2a). Adjacent live and dead microbes were presented as yellow when they were merged. Biofilm formed on denture base resin with DMADDM groups had higher dead/live microbe ratio especially for 3.3% and 6.6% DMADDM containing groups (*p* < 0.05) (Figure 2b). DMADDM containing groups also had thinner biofilm thickness compared with the control (*p* < 0.05) (Figure 2c) in line with the inhibition of multi-species biofilm (Figure 1). The metabolic activities of biofilm in DMADDM containing groups were also reduced (*p* < 0.05) (Figure 2d).

### 2.3. DMADDM Inhibited the Hypal Development and Virulence Ralated Genes Expression of C. albicans in Multi-Species Biofilms

**Figure 3:**
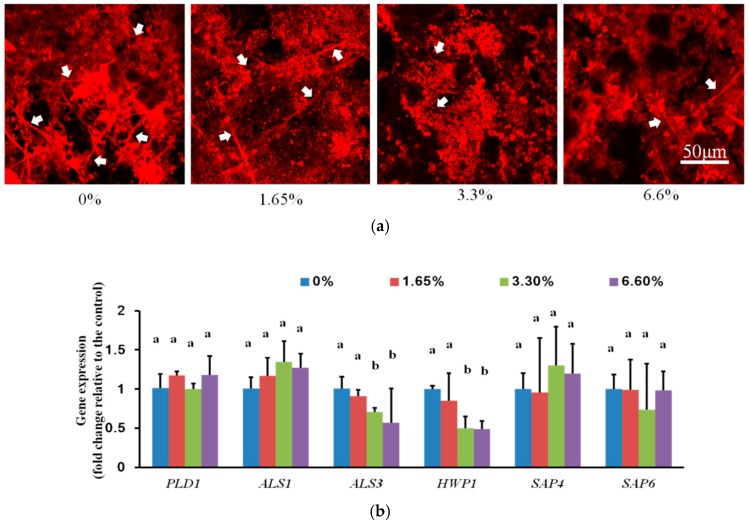
The confocal laser scanning microscope (CLSM) images and relative gene expression of *C. albicans* in biofilms: (**a**) The CLSM images of *C. albicans* in multi-species biofilms, *C. albicans* dyed red, representative hyphae form of *C. albicans* was marked with white arrows; (**b**) Relative gene expression in *C. albicans*. Values are significantly different when labelled with different letters (*p* < 0.05).

To evaluate the effect of DMADDM on *C. albicans* virulence factors in multi-species biofilm, we first observed the hyphal development of *C. albicans*. *C. albicans* was stained with concanavalin-A in multi-species biofilms (Figure 3a). Biofilms formed on DMADDM containing denture base resin specimen had less hyphae when compared with control group. Furthermore, we measured the virulence gene expression of *C. albicans* between control groups and DMADDM containing groups (Figure 3b). There were no significant expression changes among PLD1 (phospholipase D 1), ALS1 (human β-actin agglutinin-like sequence 1), SAP4 (secreted aspartyl proteinase 4), and SAP6 (secreted aspartyl proteinase 6) (*p* > 0.05) in all groups, however, ALS3 (one kind of adhesins) and HWP1 (Hyphal wall protein) were significantly downregulated (*p* < 0.05) in 3.3% and 6.6% DMADDM containing groups when compared with control group, indicating the virulence inhibitory of DMADDM containing denture base resin.

### 2.4. DMADDM Reduced the Cell Damage Caused by Multi-Species Biofilm and Also Redeced the Candida Receptor and the Pro-Flammatory Cytokine Prodction of TR-146 Cell

**Figure 4:**
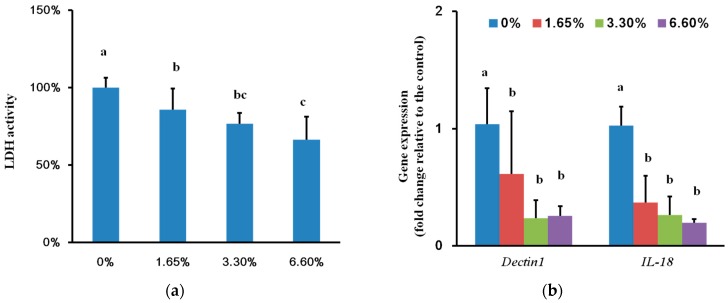
Cell damage based on lactate dehydrogenase (LDH) activity and relative gene expression of TR-146 cell: (**a**) Cell damage based on LDH activity after co-culture with biofilm formed on different DMADDM containing groups; (**b**) Relative gene expression of TR-146 cell after it was co-cultured with biofilms of different groups. Values are significantly different when labelled with different letters (*p* < 0.05).

Multi-species biofilms on DMADDM containing groups reduced the damage of TR-146 cell notably (*p* < 0.05) (Figure 4a), especially for 3.3% and 6.6% DMADDM groups (*p* < 0.05). The TR-146 cell expressed low level of IL-18 (encoding pro-inflammatory cytokine) and Dectin-1 (encoding a *Candida* receptor) when co-cultured with the biofilm formed on DMADDM containing denture base resin (*p* < 0.05) (Figure 4b) consistent with the reduced *C. albicans* virulence factors in these groups (Figure 3).

### 2.5. Biocompatibility of DMADDM Modified Denture Base Resin in Vivo

**Figure 5:**
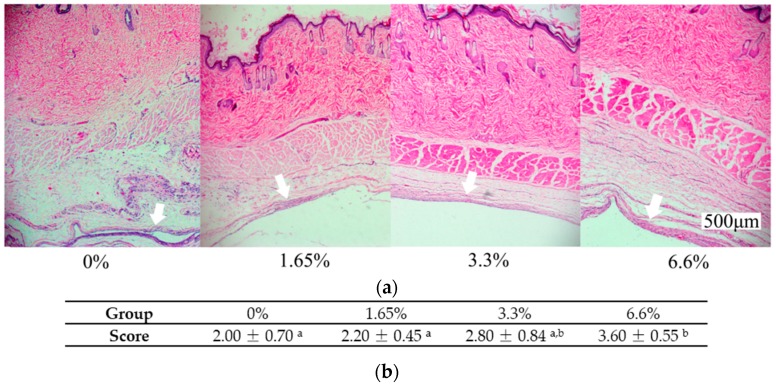
The representational images of tissue surrounded the implanted material as well as inflammatory response scores: (**a**) Representational images of tissue surrounded implanted material after eight days were all captured with a 4× objective lens and the white arrows showed the interface where tissue contacted to the material directly; (**b**) Inflammatory response scores of different groups. Values are significantly different when labelled with different letters (*p* < 0.05).

The biocompatibility of denture base resin was assessed in vivo by subcutaneous implantation in rats followed by examining the inflammation response of surrounding tissue (Figure 5a). The inflammation response of the surrounding tissue in denture base resin containing 1.65% and 3.3% DMADDM groups were similar to the control (*p* > 0.05). Only the 6.6%DMADDM containing group increased the inflammatory response (*p* < 0.05) (Figure 5b).

## 3. Discussion

The present research investigated the effect of the denture base resin containing DMADDM on multi-species biofilm as well as the biocompatibility of this new modified material. Our results confirmed that denture base resin containing DMADDM groups can significantly inhibit the multi-species biofilms containing bacteria and fungi, which not only confirmed the antibacterial ability of DMADDM as described previously [30], but also implied its antifungal ability for the first time. The less *C. albicans* in multi-species biofilm of DMADDM containing groups may result from two possible ways: (i) DMADDM can directly inhibit the growth and metabolic activity of *C. albicans* as proven by another project on *C. albicans* single species biofilm subjected to DMADDM treatment [31]; (ii) *C. albicans* dose benefit from oral bacteria [32], which means that DMADDM may also block the *C. albicans* growth promotion caused by bacterial neighbors. It was well known that QAS has the “contact killing” antibacterial mechanism [28,29], while the antifungal mechanism of QAS remained unknown. Though the cell structure of fungus is more complex than that of bacteria, the cell surface of *C. albicans* was also negative charges which may lead it to be “killed” as same as bacteria due to the “contact killing” mechanism.

*C. albicans*, an opportunistic pathogen in humans with high detection rates, is a major cause of denture stomatitis [19]. Plenty of studies used *C. albicans* single species biofilm as a model for high-throughput screening assays and antifungal testing [33,34,35]. However, bacteria also exist in denture biofilm and will undoubtedly alter the local microenvironment [7,36]. Bacteria also take part in the pathogenesis of *C. albicans* infections [37]. In this study, we used a multi-species biofilm model constructed of *C. albicans*, *S. mutans*, *A. naeslundii*, and *S. sanguinis* to stimulate the more complex denture biofilm, and to evaluate the co-effects caused by oral bacteria and *C. albicans* focusing on the phenotype of *C. albicans* which we concerned about much at the same time taking the bacteria factor into consideration [7].

It is well known that *C. albicans* hyphal form is more pathogenic than the yeast form. The hyphal development of *C. albicans* plays an important role in disease by epithelial cells invasion and tissue damage [38,39]. The *C. albicans* morphological switch from yeast form to hyphal form is not only influenced by surroundings, like temperature, composition of gas, pH, and nutrients, but also affected by the interaction with the microbiological flora [39]. Our results presented that DMADDM containing groups reduced the hyphal from in multi-species biofilm without changing the pH value (appendix B) implying that DMADDM may directly inhibit the hyphal development or disrupted the interactions between fungi and bacteria. The low expression of virulence genes *ALS3* and *HWP1* of *C. albicans* was consistent with the less hyphal form in DMADDM containing groups. *HWP1* is associated with adhesion and expresses specially on hyphae form, while *ALS3* is a kind of adhesion associated with substratum adhesion [32]. The inhibition of hyphal development together with the reduction of virulence gene expression (*HWP1* and *ALS3*) suggested that DMADDM can inhibit not only the growth of multi-species biofilm but also the virulence factors of *C. albicans*.

A recent study showed that the multi-species biofilm containing *C. albicans* and oral bacteria not only upregulated the virulence gene expression of *C. albicans* but also promoted cell damage and upregulated *IL-18* (encoding a pro-inflammatory cytokine) as well as *Dectin-1* (a *Candida* receptor) expression of reconstituted human oral epithelium after co-culture with biofilm, when compared with *C. albicans* single species biofilm [32]. Our results showed that there was lower cell damage in DMADDM containing groups (Figure 4a) and the *IL-18* and *Dectin-1* expression of TR-146 cells were also downregulated compared with the control group (Figure 4b) possibly due to the decrease of the biomass of multi-species biofilm and the inhibition of *C. albicans* virulence factors.

The well biosafety is indispensable for clinical application of materials. Previous studies used human gingival fibroblasts and odontoblast to determine the cytotoxicity in vitro and the results showed good biocompatibility with DMADDM [23,40]. A recent study showed milder pulpal inflammation in composite and adhesive containing DMADDM in a rat tooth cavity model when they investigated the antibacterial and remineralizing restorations of DMADDM [41]. In this study, we used a subcutaneous implantation to examine the tissue response of different DMADDM content groups. Our results showed that 1.65% and 3.3% DMADDM containing groups had no significant effect on inflammatory response compared with the control group, suggesting good biocompatibility and biosafety of the newly synthesized material contained DMADDM as an antimicrobial additive.

In conclusion, we granted the denture base resin the abilities of antimicrobial activities, decrease of fungal virulence factors, reduction of cell damage, with no significant change on biocompatibility by adding DMADDM until 3.3%. Though there was still a lot work to do before it could be approved for use in vivo, this novel modified resin may be considered as a new promising therapeutic system to replace the conventional denture base material placing the DMADDM containing surface to fit closely with the oral epithelium surface to combat problems caused by microbes on the denture base such as denture stomatitis.

## 4. Materials and Methods

### 4.1. DMADDM Synthesis and Preparation of Specimen

The synthesis and identification of DMADDM were according to a previous research [25] (see appendix A for detail). For specimen preparation, a heat-polymerized acrylic resin (Jianchi Dental Equipment, Changzhi, China) was used and prepared according to the specifications then made into into cube (10 mm square by 1.2 mm thick) (appendix B). For the in vitro experiment, DMADDM was mixed with one-third of specimen, at a mass fraction of 0% (control group), 1.65%, 3.3%, and 6.6% under the premise that not affected the mechanical properties [31]. For in vivo experiments, the entire specimen was mixed with DMADDM at the same mass fraction as the in vitro experiment. The surface roughness was standardized to 75.5 ± 17.5 nm using a horizontal polisher (Struers, Shanghai, China) with gradually finer aluminum oxide papers (400-, 600-, 800-, 1000-, 1200-, and 2400-grit) to exclude the roughness factor influence, as the roughness of three DMADDM containing groups had no significant difference when compared with the control group (Data not shown). To eliminate residual monomer, all the samples were immersed in distilled water for 1 day.

### 4.2. Microbial Cultures and Multi-Species Biofilm Formation

*Streptococcus sanguinis* ATCC 10556, *Candida albicans* ATCC 90028, *Actinomyces naeslundii* ATCC 12104, and *Streptococcus mutans* UA159 were all supplied by the State Key Laboratory of Oral Diseases (Sichuan University, Chengdu, China). *C. albicans* were cultured in yeast peptone dextrose (YPD) at 37 °C aerobically, while *S. mutans*, *S. sanguinis*, and *A. naeslundii* were cultured in brain heart infusion broth (BHI; Oxoid, Basingstoke, UK) with the condition of 37 °C anaerobically (5% CO_2_, 90% N_2_, 5% H_2_).

The final inoculum for inter-kingdom biofilm formation was composed of *C. albicans* (10^5^ colony forming unit (CFU)/mL) and bacteria (10^7^ CFU/mL each) in 2 mL McBain medium [42] with 50 mM piperazine-1,4-bisethanesulfonic acid (PIPES) and 0.2% sucrose in 24-well plates with specimens placed at 37 °C aerobically for 72 h and the media was changed every 24 h. The final microbial content of inoculum was determined according to previous research [32].

### 4.3. pH Measurement

For pH measurement, the 2 mL supernatant of biofilms was collected and measured by pH meter (Thermo Scientific, Waltham, MA, USA) at 24, 48, and 72 h (appendix C).

### 4.4. Colony-Forming Unit (CFU) Counts

After culture, specimens with 72 h biofilms were washed twice with PBS buffer to remove planktonic microbes then moved to culture dishes with 2 mL phosphate buffer saline (PBS) buffer in advance. Biofilms were collected using sterilized blades to remove microbes from the specimen then vortexed thoroughly. Brain heart infusion broth (BHI) agar plates and Sabouraud’s dextrose agar (SDA) were prepared for culturing the microbes which were gradient diluted in PBS previously. Then BHI and SDA agar plates were cultured aerobically at 37 °C for 2 days for CFU counting.

### 4.5. 2,3-Bis(2-methoxy-4-nitro-5-sulfophenyl)-2H-tetrazolium-5-carboxanilide (XTT) Array

XTT array were conducted to measure the metabolic activity of biofilm as described previously [43]. Specimen with biofilm was washed using PBS followed by adding 1 mL XTT working regent (XTT at concentration of 0.5 g/L with menadione at concentration of 1 μM) then cultured for 2 h at 37 °C. Microplate spectrophotometer (Gene, Hong Kong, China) was used to read the OD_490_ nm.

### 4.6. Cell Culture

The commercially obtained TR-146 cell (JENNIO Biological Technology, Guangzhou, China) was routinely cultured in dulbecco’s modification of eagle’s medium (DMEM) (Gibco, Carlsbad, CA, USA) + 10% FBS (fatal bovine serum, Gibco) at 37 °C and 5% CO_2_. 12 mm diameter glass coverslips were placed at the bottom of 12-well plates. Then the cells were seeded into the plate and cultured until confluency was reached.

### 4.7. Cell Damage Assay

LDH array were conducted to determine the TR 146 cell damage after the cells were co-cultured with biofilm as previously [32,44]. Briefly, monolayers of TR-146 cell grown on glass were overlaid on biofilm’s surface which formed on acrylic coupons then incubated for 6 h in 2 mL FBS-free DMEM with the condition of 37 °C and 5% CO_2_. After that, the supernatant was used for LDH array while the TR-146 cell was used for RNA isolation for Real-time PCR. The LDH activity was analyzed using a Roche cytotoxicity detection kit^plus^ (LDH) followed by measuring absorbance at 492 nm. For background cell control, after monolayer TR-146 cells were incubated with DMEM, the supernatant was used for LDH activity detection. For background biofilm control, after biofilm was incubated with DMEM, the supernatant was used for LDH activity detection. For high control, 1% Triton X-100 was added by using a pipette tip to disrupt monolayer TR-146 cells for the last 1 h of incubation. The cytotoxicity percentage was determined in the following equation: experimental value minus cells control minus biofilm control/mean high control minus cells control.

### 4.8. RNA Isolation and Real-Time PCR

For biofilm’s RNA isolation, the microbes were collected by centrifugation then were resuspended in RNALater (Life Technologies, Carlsbad, CA, USA). The samples were stored at −20 °C until RNA extraction. Microorganisms were resuspended in 1 mL TRIzol regent (Invitrogen, Carlsbad, CA, USA) and then were disrupted in a Precellys 24 system (Bertin Technologies, Paris, France) by high-speed homogenization with glass beads. Phenol:chloroform:isoamyl alcohol (25:24:1) (Solorbio, Beijing, China) was used for demixing to acquire total nucleic acid. For RNA isolation from TR-146 cell after it was co-cultured with biofilm, total RNA was isolated form cells using the TRizol regent (Invitrogen) according to manufacturers’ instructions. Then the DNA contamination from two kinds of samples was removed by PrimeScript™ RT reagent Kit with gDNA Eraser (Perfect Real Time) from Takara Bio (Otsu, Japan). The total RNA concentration and purity from two kinds of samples were measured by a Nanodrop 2000 spectrophotometer from Thermo Scientific. Reverse transcription was performed by using a PrimeScript™ RT reagent Kit with gDNA Eraser (Perfect Real Time) from Takara Bio.

Triplicate Real-time PCRs were performed in 96-well plates in CFX 96 equipment (Bio-Rad, Hercules, CA, USA) using SYBR^®^ Premix Ex Taq™ (Tli RNaseH Plus) from Takara Bio. 20 μL of each reaction system comprised 10 μL SYBR Premix Ex Taq II (2×), 2 μL template cDNA, and forward and reverse primers (10 μM each list in appendix D, Table A1). *ACT1* was used to normalize the gene expression of *C. albicans* while the *β-actin* as was used as the reference gene for TR-146 cell. The reaction conditions are set as follows: 2 min at 95 °C, then 40 cycles of 15 s at 95 °C, and 30 s at 58 °C. Then the results calculated by normalizing target genes to respective reference gene based on the 2^−ΔΔ*C*t^ method.

### 4.9. Biofilm Imaging

Live/dead staining was used for observing the microbial viability. For live/dead imaging, the biofilms were stained with 2.5 μM SYTO 9 and propidium iodide (Invitrogen) for 15 min following the manufacturer’s instruction [32]. For *Candida* staining, the biofilm were stained with 25 μM concanavalin-A conjugated with Alexa Fluor^®^ 594 (Invitrogen) for 30 min [45]. The Leica CLSM was used to acquire the biofilms images using a 63× objective lens. The channels were set as follows: excitation/emission maxima 480/500 nm for SYTO 9 stain, 490/635 nm for propidium iodide stain, while 590/617 nm for concanavalin-A stain.

All the biofilms’ three-dimensional reconstruction and thickness were performed by software Imaris 7.0.0 (Bitplane, Zürich, Switzerland). The live/dead bacteria biomass was quantified using software COMSTAT (http://www.imageanalysis.dk) as described previously [25,46].

### 4.10. Biocompatibility Experiment

The toxicity of denture base resin containing DMADDM was operated as described before [47]. Briefly, 10-week-old male Wistar rats with starting body weights of 285–300 g were obtained from Chengdu Dashuo Biotechnology Co., Ltd. (Chengdu, China). Specimens that contained various concentrations of DMADDM were subcutaneously implanted into Wistar rats. Ten male Wistar rats were separated into two groups. Five Wistar rats were implanted into one piece of 0% DMADDM specimen (control) on the left side and one piece of 1.65% DMADDM specimen on the right side. Another 5 Wistar rats were implanted into one piece of 3.3% DMADDM specimen on the left side and one piece of 6.6% DMADDM specimen on the opposite side. After 8 days, all the rats were anesthetized to sacrifice. 4% paraformaldehyde-fixed tissue surrounding specimen was used for histopathological analysis. 5 μm paraffin-embedded tissue sections were prepared with HE staining technique and HE images all captured with a 4× objective lens. Tissue inflammatory response was evaluated according to 5 fields with each group assigned severity grades according to none or few, slight, moderate, and severe inflammatory level as a previous study which scored 1–4, respectively [37]. The animal experiment was conducted following the guiding principles of local Animal Welfare Committee (Ethics Committee of West China Hospital of Stomatology, Sichuan University， Chengdu, China) (registration no.WCCSIRB-2015-108, date of approval: 22 October 2015).

### 4.11. Statistical Analysis

Data was subjected to one-way ANOVA to examine the significance of variables followed with Student–Newman–Keuls except the inflammatory response scores. Differences were considered significant when *p* < 0.05. While inflammatory response scores were subjected to nonparametric Kruskall–Wallis analysis and the Mann–Whitney U-test at α = 0.05 level. Software SPSS16.0 (SPSS Inc., Chicago, IL, USA) was operated for statistical analysis.

## Figures and Tables

**Figure B1:**
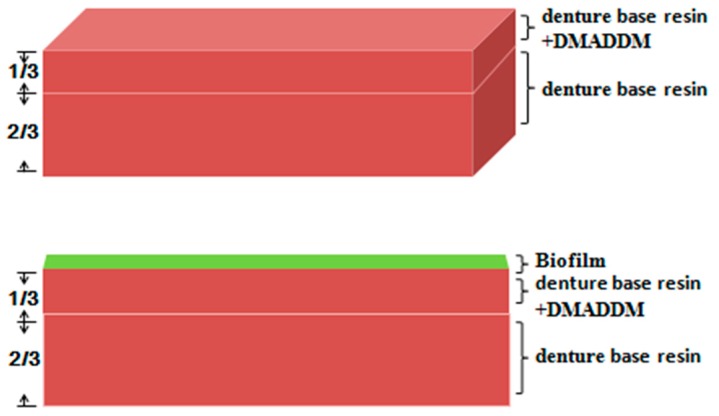
The specimen model for all experiments except for animal experiment.

**Figure C1:**
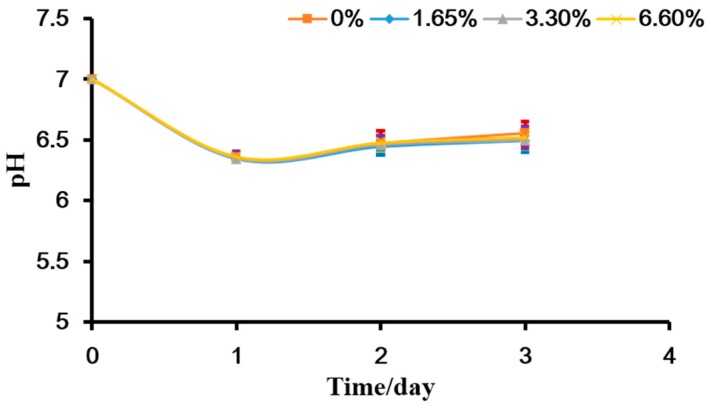
The 2 mL supernatant pH during biofilms formation (There were no significant difference in all groups).

## Abbreviations

DMADDM	Dimethylaminododecyl methacrylate
QAS	Quaternary ammonium salts
DMAD	1-(dimethylamino)dodecan
BEMA	2-bromoethyl methacrylate
CLSM	Confocal laser scanning microscope
LDH	Lactate dehydrogenase

## Appendix A

### Synthesis of DMADDM

Briefly, 10 mmol of 1-(dimethylamino)dodecan (DMAD) (Tokyo Chemical Industry, Tokyo, Japan), 10 mmol of 2-bromoethyl methacrylate (BEMA) and 3 g ethanol were added in a 20 mL vial with a magnetic stir bar. The vial was capped and stirred at 70 °C for 24 h. After that, the ethanol solvent was removed via evaporation, yielding DMADDM as a clear, colorless, and viscous liquid.

## Appendix D

**Table D1:** Primers used in Real-time PCR [31]. FW: forward; RV: reverse.

Primers	Nucelotide Sequence (5′–3′)
*ACT1*	FW-TGCTGAACGTATGCAAAAGG
	RV-TGAACAATGGATGGACCAGA
*ALS1*	FW-CCCAACTTGGAATGCTGTTT
	RV-TTTCAAAGCGTCGTTCACAG
*ALS3*	FW-CTGGACCACCAGGAAACACT
	RV-GGTGGAGCGGTGACAGTAGT
*SAP4*	FW-GTCAATGTCAACGCTGGTGTCC
	RV-ATTCCGAAGCAGGAACGGTGTCC
*SAP6*	FW-AAAATGGCGTGGTGACAGAGGT
	RV-CGTTGGCTTGGAAACCAATACC
*HWP1*	FW-TCTACTGCTCCAGCCACTGA
	RV-CCAGCAGGAATTGTTTCCAT
*PLD1*	FW-GCCAAGAGAGCAAGGGTTAGCA
	RV-CGGATTCGTCATCCATTTCTCC
*β-actin*	FW-GAGCACAGAGCCTCGCCTTTGCCGAT
	RV-ATCCTTCTGACCCATGCCCACCATCACG
*IL-18*	FW-CCTTCCAGATCGCTTCCTCTCGCAACAA
	RV-CAAGCTTGCCAAAGTAATCTGATTCCAGGT
*Dectin1*	FW-ACAGCAATGAGGCGCCAAGGAGGAGATG
	RV-GGAGCAGAAAGAAAAGAGCTCCCAAATGCT
